# Investigation of Antidiabetic, Antihyperlipidemic, and *In Vivo* Antioxidant Properties of *Sphaeranthus indicus* Linn. in Type 1 Diabetic Rats: An Identification of Possible Biomarkers

**DOI:** 10.1155/2011/571721

**Published:** 2010-09-26

**Authors:** S. Ramachandran, K. Asokkumar, M. Uma Maheswari, T. K. Ravi, A. T. Sivashanmugam, S. Saravanan, A. Rajasekaran, J. Dharman

**Affiliations:** ^1^Department of Pharmacology, KMCH College of Pharmacy, Tamil Nadu, Coimbatore 641 048, India; ^2^Department of Pharmacology, College of Pharmacy, SRIPMS, Tamil Nadu, Coimbatore 641 044, India; ^3^Department of Pharmaceutical Analysis, College of Pharmacy, SRIPMS, Tamil Nadu, Coimbatore 641 044, India; ^4^Department of Pharmacology, PSG College of Pharmacy, Tamil Nadu, Coimbatore 641 004, India; ^5^Department of Pharmaceutical Chemistry, KMCH College of Pharmacy, Tamil Nadu, Coimbatore 641 048, India; ^6^Department of Pharmaceutical Analysis, KMCH College of Pharmacy, Tamil Nadu, Coimbatore 641 048, India

## Abstract

The present investigation was aimed to study the antidiabetic, antihyperlipidemic, and *in vivo* antioxidant properties of the root of *Sphaeranthus indicus* Linn. in streptozotocin- (STZ-) induced type 1 diabetic rats. Administration of ethanolic extract of *Sphaeranthus indicus* root (EESIR) 100 and 200 mg/kg to the STZ-induced diabetic rats showed significant (*P* < .01) reduction in blood glucose and increase in body weight compared to diabetic control rats. Both the doses of EESIR-treated diabetic rats showed significant (*P* < .01) alteration in elevated lipid profile levels than diabetic control rats. The EESIR treatment in diabetic rats produced significant increase in superoxide dismutase (SOD), catalase (CAT), glutathione peroxidase (GPx) and decrease in thiobarbituric acid reactive substances (TBARS) levels than diabetic control rats. Administration of EESIR 200 mg/kg produced significant (*P* < .01) higher antioxidant activity than EESIR 100 mg/kg. The high performance liquid chromatography (HPLC) analysis of EESIR revealed the presence of biomarkers gallic acid and quercetin. In conclusion, EESIR possess antidiabetic, antihyperlipidemic, and *in vivo* antioxidant activity in type 1 diabetic rats. Its antioxidant and lipid lowering effect will help to prevent diabetic complications, and these actions are possibly due to presence of above biomarkers.

## 1. Introduction

Diabetes mellitus (DM) is a group of metabolic diseases characterized by hyperglycemia resulting from the defects in insulin secretion, insulin action, or both. A chronic hyperglycemic condition in diabetes is associated with long-term damage, dysfunction, and failure of various organs, such as eyes, kidneys, nerves, heart, and blood vessels. It is the most common serious metabolic disorder and is considered to be one of the five leading causes of death in the world [1]. DM is classified into two major categories: type 1 and type 2 diabetes. Although both types of diabetes have distinct pathogenesis, hyperglycemia, and various life-threatening complications are common to both [2]. The plasma lipids are usually raised in diabetes and such an elevation represents a risk factor for coronary heart disease [3]. Free radicals have significant role in the causation of several diseases like diabetes, parkinson's disease, cirrhohsis, cancer, and cardiovascular disorders [4]. In DM, oxidative damage and tissue injury may lead to alterations in the endogenous free radical scavenging defense mechanisms and ineffective scavenging of reactive oxygen species [5]. The current treatment for control of DM includes diet, exercise, oral antidiabetic drugs, and insulin therapy. However, insulin and other oral hypoglycemic drugs have characteristic profile of adverse effects. This has initiated the identification of novel drugs which might act in mechanistically distinct way compared to existing drug targets [6]. Hence, research is focused on medicinal plants which are used in the traditional practices and development of newer drug leads from phytoconstituents with more potential and effective agents with lesser side effects than the existing hypoglycemic agents [1]. Traditionally, many medicinal plants are currently used in India for the treatment of diabetes and its efficacy has been proved scientifically [7]. 

*Sphaeranthus indicus* Linn. (Family-Compositae) is a branched herb with purple flowers that grows abundantly in rice field and distributed throughout India. It is used indigenously in the Indian system of medicine as an anthelmintic [8]. The plant has a wide range of medicinal value and has been used in hemicranias, jaundice, leprosy, diabetes, fever, pectoralgia, cough, gastropathy, hernia, hemorrhoids, helminthiasis, dyspepsia, skin diseases and nerve tonic [9, 10]. Pharmacological activities such as immunomodulatory [11], antimicrobial [12, 13], antibacterial [14, 15], anxiolytic [10], wound healing action [16] were reported on this plant. Phytoconstituents isolated from this plant are eudesmanolides [17], isoflavonoids [18], 7-hydroxy eudesmanolides [19], sterol glycoside [20], essential oil (cadiene, ocimene, citral, p-methoxycinnamaldehyde, geraniol, eugenol and geranyl acetate) [21], and eudesmanolides [22]. In India, the tribals of Madhya Pradesh used this plant for the treatment of diabetes [23]. Globally, type 1 DM affects considerable percentage of population and it leads to morbidity and mortality of the diabetic patients. Antioxidants play a major role in the prevention of diabetes and its complications by scavenging free radicals. Survey of current literature revealed that there is no scientific data documented for the effect of *Sphaeranthus indicus* root in the treatment of type 1 diabetes mellitus and its *in vivo* antioxidant action. Therefore, the present study was undertaken to investigate the antidiabetic, antihyperlipidemic and *in vivo* antioxidant potential of EESIR in type 1 diabetic rats as well as identification of the possible biomarkers in EESIR.

## 2. Methods

### 2.1. Plant Material

*Sphaeranthus indicus* Linn. was collected from Erode, Tamil Nadu, India and authenticated by Dr.P.Venu, Joint Director of Botanical Survey of India, Tamil Nadu Agriculture University (TNAU), Coimbatore. The authentication certificate bearing number BSI/SC/5/21/04-05/Tech-1850 is documented in our laboratory. The roots were separated, cleaned, air dried under shade and powdered using mechanical grinder and stored in air-tight container.

### 2.2. Preparation of EESIR

Dried powdered plant roots were (85 g) extracted with 70% v/v ethanol using Soxhlet apparatus for 72 hours. The extract was concentrated under reduced pressure in rota evaporator and yield of the extract was found to be 15.24 g. The EESIR was stored at 10°C in the refrigerator until the completion of pharmacological studies.

### 2.3. Drugs and Chemicals

Acetonitrile: n-hexane sulphonic acid were of HPLC grade (Himedia, Mumbai). Gallic acid and quercetin reference standards were purchased from Sigma Aldrich (USA). Streptozotocin was procured from Sisco Research Laboratories Private Ltd., Mumbai, India. All other chemicals and reagents were of analytical grade, and enzymatic kits used in this study were obtained commercially.

### 2.4. Preparation of Standard and Sample Solutions for HPLC

Stock solutions of gallic acid (GA) and quercetin (QA) were prepared at concentration of 1000 *μ*g/ml and 2000 *μ*g/ml immediately before use and used as reference standard. The EESIR was dissolved in mobile phase, to obtain a concentration of 4 mg/ml and used as sample solution. All sample solutions were filtered through 0.45 *μ*m membrane filter (Millipore) and injected directly. 

### 2.5. Chromatographic Conditions for HPLC

Chromatographic analysis was carried out by Phenomenex-Luna C18 reversed-phase column (ø 250 mm × 4.6 mm) packed with 5 *μ*m diameter particles; the mobile phase was acetonitrile: n-hexane sulphonic acid (20 : 80 v/v) pH 2.5 adjusted with glacial acetic acid. This mobile phase was filtered through a 0.45 *μ*m membrane filter (Millipore), then deaerated ultrasonically prior to use. The GA and QA were quantified by photodiode array detector following HPLC separation at 299 nm. The mobile phase flow rate and injection volume were 1.2 ml/min and 20 *μ*L, respectively. The chromatographic peaks of the analytes were confirmed by comparing their retention time (Rt) and UV spectra with those of the reference standards. The GA (2.5 to 12.5 *μ*g/ml) and QA (10 to 80 *μ*g/ml) standard solutions were injected into the HPLC and peak area responses obtained. Standard graphs were prepared by plotting concentration versus area. Quantification was carried out from integrated peak areas of the samples using the corresponding standard graph. All chromatographic operations were carried out at ambient temperature.

### 2.6. Experimental Animals

*Wistar *albino rats of both sexes weighing 150–200 g were used for the study. All animals were maintained under standard laboratory conditions [temperature (22 ± 2°C) and humidity (45 ± 5°C)] with 12 hours day: 12 hours night cycle. The animals were fed with normal laboratory diet and allowed to drink water *ad libitum*. All the experimental procedures conducted after the approval of ethical committee (817/04/ac/CPCSEA) and were in strict accordance with institutional animal ethical committee guidelines for the care and use of laboratory animals.

### 2.7. Acute Toxicity

Acute oral toxicity study was performed as per Organization for Economic Cooperation and Development (OECD) guidelines 423 [24]. After the oral administration of EESIR, animals were observed individually at least once during the first 30 minutes and periodically during the first 24 hours, with special attention given during the first 4 hours, and daily thereafter, for a total of 14 days.

### 2.8. Induction of Diabetes

Diabetes was induced in overnight fasted rats by intraperitoneal injection of STZ at a dose of 60 mg/kg body weight [25] in 0.1 M cold citrate buffer (pH 4.5). STZ can induce fatal hypoglycemia as a result of massive pancreatic insulin release and to avoid this hypoglycemic effect, the rats were provided with 5% dextrose solution after 6 hours of STZ administration for next 24 hours. Induction of diabetes was verified after 72 hours and the animals were allowed 14 days for the stabilization of blood glucose level. At 14th day, animals having a blood glucose level higher than 210 mg/dl were considered diabetic and used for the experiments.

### 2.9. Experimental Design for Antidiabetic Activity

The rats were divided into five groups of six each randomly: group 1 normal untreated rats which received 0.2% carboxy methyl cellulose (CMC) served as normal control, group 2 diabetic rats received 0.2% CMC served as diabetic control, group 3 diabetic rats received EESIR (100 mg/kg), group 4 diabetic rats received EESIR (200 mg/kg), and group 5 diabetic rats received glibenclamide (5 mg/kg). The EESIR and glibenclamide was suspended in 0.2% CMC and administered orally for 28 days once daily to the respective groups. On the 28th day, the animals were fed with respective dose of drug and, the body weights of all the animals were determined and blood samples were collected after 1 hour post drug dose. The serum was separated by centrifugation of blood at 5000 rpm for 10 minutes and the biochemical parameters were analyzed.

### 2.10. Estimation of Glucose and Lipid Profile Levels

The blood glucose level was determined by glucose oxidase-peroxidase method using glucose enzymatic kit (Agappe diagnostic, Kerala, India) and expressed in milligrams per deciliter (mg/dl). The lipid profiles such as total cholesterol (TC), triglycerides (TG), high density lipoprotein (HDL) were determined using enzymatic kits (Agappe diagnostic, Kerala, India) and low density lipoprotein (LDL), very low density lipoprotein (VLDL) values were calculated by Friedewalds formula as given below [26]

(1) VLDL=TG/5,

(2) LDL=TC−(HDL+VLDL).

### 2.11. Determination of Lipid Peroxidation Indices and Antioxidant Levels

Liver was dissected out and washed immediately with ice cold saline to remove blood. Tissue homogenate (10% w/v) was prepared with 0.025 M Tris–HCl buffer (pH 7.5) and was used for the assay of lipid peroxidation [27], superoxide dismutase [28], catalase [29], and glutathione peroxidase [30].

### 2.12. Statistical Analysis

All the data are expressed as mean ± SEM and evaluated by one way analysis of variance (ANOVA), employing Dunnett's test for multiple comparisons and values of *P* < .05 were considered as statistically significant.

## 3. Results

### 3.1. HPLC Estimation

**Figure 1:**
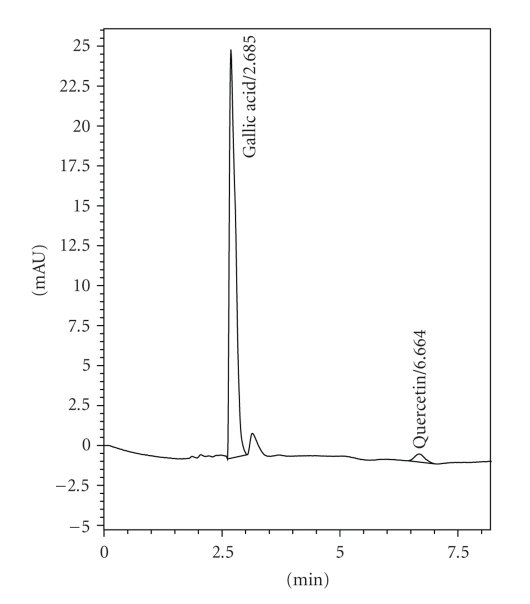
HPLC chromatogram of standard gallic acid and quercetin.

**Figure 2:**
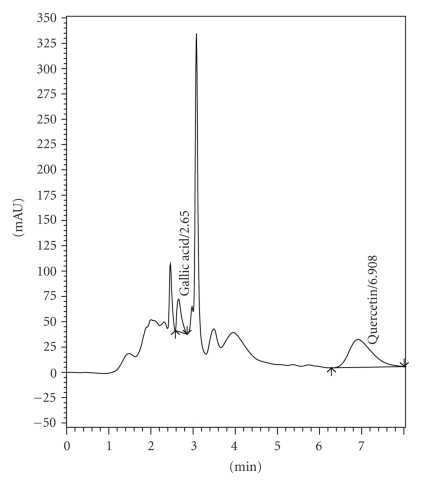
HPLC chromatogram of EESIR for gallic acid and quercetin.

The HPLC fingerprints of the EESIR showed two types of the phytoconstituents, gallic acid, and quercetin. The retention time of standard GA and QA were found to be 2.685 and 6.664 (Figure 1). The retention time of GA and QA in EESIR was found to be 2.650 and 6.908 (Figure 2), and it matched with standard Rt values, respectively. The amount of GA and QA in EESIR was found to be 1.92 mg/g and 216 mg/g of extract.

### 3.2. Determination of Acute Toxicity, Body Weight, and Glucose Levels

**Figure 3:**
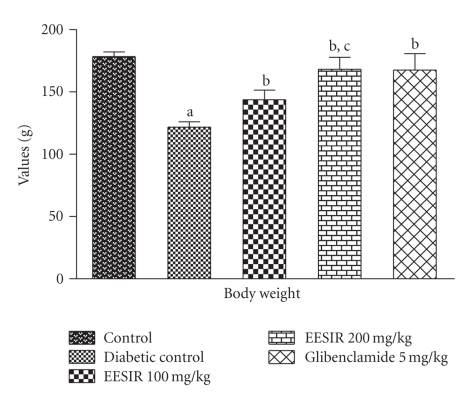
Effect of ethanolic extract of *Sphaeranthus indicus* root on body weight in STZ-induced diabetic rats. All data are expressed in mean ± SEM (*n* = 6). ^a^ *P* < .01: diabetic control group compared with normal control group. ^b^ *P* < .01: EESIR 100, 200 mg/kg and glibenclamide 5 mg/kg compared with diabetic control group. ^c^ *P* < .01: EESIR 200 mg/kg compared with EESIR 100 mg/kg group.

**Figure 4:**
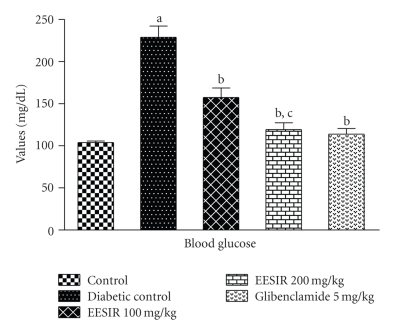
Effect of ethanolic extract of *Sphaeranthus indicus* root on blood glucose in STZ-induced diabetic rats. All data are expressed in mean ± SEM (*n* = 6). ^a^ *P* < .01: diabetic control group compared with normal control group. ^b^ *P* < .01: EESIR 100, 200 mg/kg and glibenclamide 5 mg/kg compared with diabetic control group. ^c^ *P* < .01: EESIR 200 mg/kg compared with EESIR 100 mg/kg group.

The oral administration of EESIR at the dose 2000 mg/kg did not exhibit death and any signs of toxicity up to 14 days (LD_50_ > 2000 mg/kg). Therefore, the biological evaluation was carried out using 100 and 200 mg/kg dose levels. The body weight of the diabetic rats showed significant (*P* < .01) reduction after the administration of STZ compared to normal control rats. The treatment of EESIR at a dose 100 and 200 mg/kg showed a significant (*P* < .01) increase in body weight compared to diabetic control rats. The effect of EESIR at 200 mg/kg dose level on body weight is almost equal to that of glibenclamide (Figure 3). Both the doses of EESIR showed significant (*P* < .01) reduction in elevated blood glucose level compared to diabetic control group. The administration of EESIR 200 mg/kg produced significant (*P* < .01) decrease in blood glucose level than 100 mg/kg, and however, it was found less effective than the standard drug glibenclamide in controlling elevated blood glucose level (Figure 4).

### 3.3. Alteration of Lipid Profile by EESIR 

**Figure 5:**
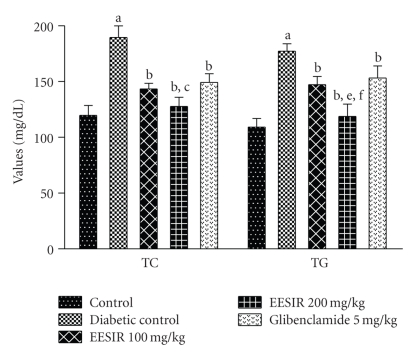
Effect of ethanolic extract of *Sphaeranthus indicus* root on TC and TG in STZ-induced diabetic rats. All data are expressed in mean ± SEM (*n* = 6). ^a^ *P* < .01: diabetic control compared with normal control group. ^b^ *P* < .01: EESIR 100, 200 mg/kg and glibenclamide 5 mg/kg compared with diabetic control group. ^c^ *P* < .05: EESIR 200 mg/kg compared with EESIR 100 mg/kg group. ^e^ *P* < .01: EESIR 200 mg/kg compared with glibenclamide 5 mg/kg group. ^f^ *P* < .01: EESIR 200 mg/kg compared with EESIR 100 mg/kg group.

**Figure 6:**
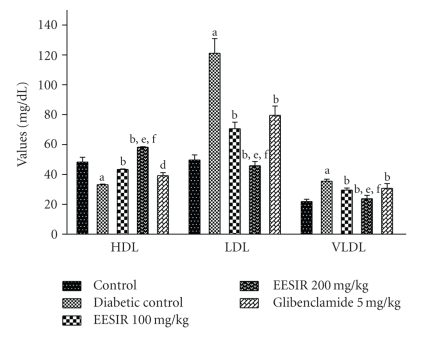
Effect of ethanolic extract of *Sphaeranthus indicus* root on HDL, LDL, and VLDL in STZ-induced diabetic rats. All data are expressed in mean ± SEM (*n* = 6). ^a^ *P* < .01: diabetic control compared with normal control group. ^b^ *P* < .01: EESIR 100, 200 mg/kg and glibenclamide 5 mg/kg compared with diabetic control group. ^d^ *P* < .05: glibenclamide 5 mg/kg compared with diabetic control group. ^e^ *P* < .01: EESIR 200 mg/kg compared with glibenclamide 5 mg/kg group. ^f^ *P* < .01: EESIR 200 mg/kg compared with EESIR 100 mg/kg group.

In this study, after administration of STZ, profound alterations of the lipid profile were seen in diabetic rats. Both the doses of EESIR showed significant (*P* < .01) reduction in elevated TC, TG, and LDL levels and increased HDL level. The dose of 200 mg/kg showed significant (*P* < .05 and *P* < .01) higher reduction in elevated TC,TG, and LDL levels when compared to glibenclamide and 100 mg/kg dose of EESIR. The HDL level at the dose of EESIR 200 mg/kg was significantly increased compared to glibenclamide and EESIR 100 mg/kg (*P* < .01). Administration of EESIR and glibenclamide to diabetic rats showed significant (*P* < .01) reduction in VLDL level than diabetic control (Figures 5 and 6).

### 3.4. Antioxidant Action in Liver Homogenate

**Figure 7:**
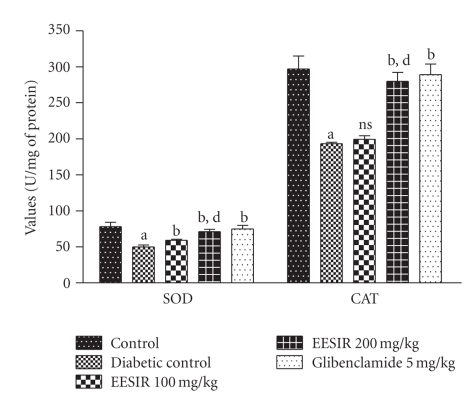
Effect of ethanolic extract of *Sphaeranthus indicus* root on SOD and CAT in STZ-induced diabetic rats. All data are expressed in mean ± SEM (*n* = 6). ^a^ *P* < .01: diabetic control compared with normal control group. ^b^ *P* < .01: EESIR 100, 200 mg/kg and glibenclamide 5 mg/kg compared with diabetic control group. ^d^ *P* < .01: EESIR 200 mg/kg compared with EESIR 100 mg/kg group. ns: No significance.

**Figure 8:**
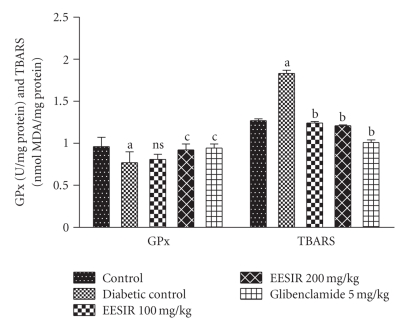
Effect of ethanolic extract of *Sphaeranthus indicus* root on GPx and LPO in STZ-induced diabetic rats. All data are expressed in mean ± SEM (*n* = 6). ^a^ *P* < .01: diabetic control compared with normal control group. ^b^ *P* < .01: EESIR 100, 200 mg/kg and glibenclamide 5 mg/kg compared with diabetic control group. ^c^ *P* < .05: EESIR 200 mg/kg and glibenclamide 5 mg/kg compared with diabetic control group. ns: No significance.

There was a significant reduction of SOD, GPx, and CAT and elevation of TBARS levels were observed in diabetic rats compared to control animals. The administration of EESIR (*P* < .01 and *P* < .05) significantly increased the SOD, GPx, CAT and reduced TBARS level in dose-dependant manner (Figures 7 and 8). However, the dose of 200 mg/kg dose of EESIR significantly (*P* < .01) increased SOD and CAT level when compared to 100 mg/kg dose group. Moreover, diabetic rats treated with EESIR 200 mg/kg showed equal efficacy to improve antioxidant level compared to standard drug glibenclamide.

## 4. Discussion

The administration of STZ produces selective pancreatic islet beta cell cytotoxicity and has been commonly used to produce type 1 diabetes in the experimental animal model [31]. The loss of body weight after administration of STZ may be due to the loss of degradation of structural proteins [32]. The administration of EESIR improves body weight compared to diabetic rats and it indicates preventive effect of EESIR on degradation of structural proteins. The massive destruction of pancreatic beta cells after STZ injection is due to alkylation of DNA thereby producing hyperglycaemia [33], and it accounts for drastic reduction in insulin level which in turn alters glucose utilization and metabolism. The medicinal plants having hypoglycemic activity act through multiple mechanisms such as improving insulin sensitivity, augmenting glucose-dependent insulin secretion, and stimulating the regeneration of islets of langerhans in pancreas of STZ-induced diabetic rats [34]. After the administration of EESIR, significant reduction in elevated glucose level was observed in diabetic rats, and it may be due to the action mediated by EESIR in any one of the above mechanism. 

The risk of coronary heart disease risk (CHD) is increased in DM due to profound alterations in the plasma lipids and lipoprotein profile [35, 36]. The elevated level of total cholesterol is one of the major factors for occurrence of CHD. Also, altered lipid profile levels and the incidence of atherosclerosis are increased in DM [37]. Hence, control or reduction of lipid profiles level in diabetic condition would reduce mortality rate. Many medicinal plants and herbs reported to possess antihyperlipidemic and antidiabetic effect [38, 39]. The blood glucose and lipid lowering efficacy of EESIR could be beneficial in preventing diabetic-related complications and to improve compliance of diabetic patients. 

Oxidative stress plays a major role in generation of free radicals in the pathogenesis of diabetes and its complications. In the pathogenesis of diabetic complication, oxidative processes mediated by free radicals have major role. Autoxidation of glucose and nonenzymatic protein glycation occurs during persistent hyperglycemia, and this may cause disruption of cellular function and oxidative damage of cell membranes due to increased level of free radicals. The cell components such as lipid, protein, DNA, and carbohydrates are affected by free radicals. The lipids are affected by higher level than protein and carbohydrates [40]. In present study, oxidative stress induced by STZ may lead to disturbance of *in vivo* antioxidant system. The level of lipid peroxidation (TBARS) and reactive oxygen species (superoxide anion, hydrogen peroxide, and hydroxyl radical) are common markers of oxidative stress in diabetic rats. An increased level of TBARS was observed in diabetic rats and the administration of EESIR significantly reduced TBARS levels. The SOD catalyses the conversion of superoxide anion to hydrogen peroxide and oxygen [41]. In diabetic rats, liver SOD level was reduced on comparison to normal control and after EESIR treatment there was a significant increase in SOD level. CAT is a hemoprotein enzyme and it catalyzes the reduction of hydrogen peroxide and protects tissues from highly reactive hydroxyl radicals [42]. The level of CAT was reduced in diabetic rats and administration of EESIR improved CAT level in diabetic rats. In normal condition, GPx detoxifies hydrogen peroxide to water through the oxidation of reduced glutathione [43]. The decreased level of GPx activity in diabetic control rats indicates an important adaptive response to increased peroxidative stress [44]. The administration of EESIR showed increased GPx activity when compared to diabetic control rats. The ability of EESIR to restore the antioxidant status in diabetic rats clearly indicates its free radical scavenging property. The present study provides more scientific support to the *in vitro* report [45]. 

**Figure 9:**
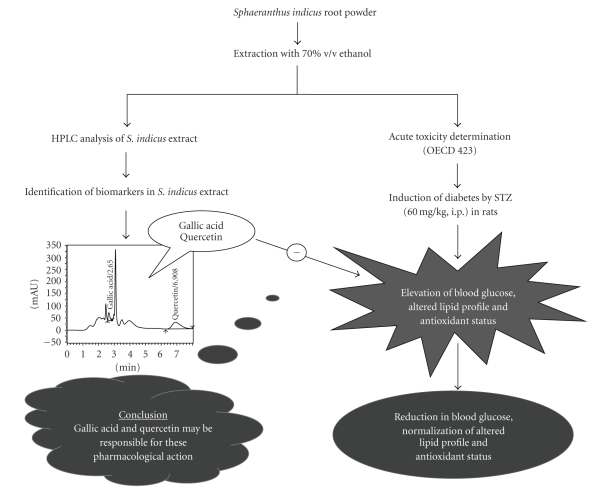
Schematic diagram.

The HPLC chromatogram confirms the presence of biomarkers such as gallic acid and quercetin in EESIR. The insulin releasing capacity of quercetin in isolated rat islets of langerhans is well documented [46]. The antioxidant property of gallic acid has been reported [47] and enhancement of insulin receptor sensitivity by gallic acid may be responsible for its antidiabetic action [48]. Moreover, the content of quercetin in EESIR was higher than gallic acid, and hence this data supports that quercetin may be responsible for the antidiabetic, antihyperlipidemic, and antioxidant activity. The gallic acid may act adjuvant with quercetin that probably results in better pharmacological action of EESIR. The hypothetical pharmacological action of *S. indicus* root is represented in the schematic diagram (Figure 9).

## 5. Conclusion

The present findings clearly demonstrated that the roots of *Sphaeranthus indicus *exhibited antidiabetic, hypolipidemic, and *in vivo* antioxidant effects in STZ-induced diabetes. These activities may possibly due to presence of biomarker compounds such as gallic acid and quercetin in this plant root which indirectly helped to decrease the levels of glucose, prevent the alteration of lipids level and increase antioxidant status in diabetic condition.
